# Variations in Rodent Models of Type 1 Diabetes: Islet Morphology

**DOI:** 10.1155/2013/965832

**Published:** 2013-05-13

**Authors:** Lesya Novikova, Irina V. Smirnova, Sonia Rawal, Abby L. Dotson, Stephen H. Benedict, Lisa Stehno-Bittel

**Affiliations:** ^1^Department of Physical Therapy and Rehabilitation Science, University of Kansas Medical Center, Kansas City, KS 66160, USA; ^2^Department of Molecular Biosciences, University of Kansas, Lawrence, KS 66045, USA

## Abstract

Type 1 diabetes (T1D) is characterized by hyperglycemia due to lost or damaged islet insulin-producing **β**-cells. Rodent models of T1D result in hyperglycemia, but with different forms of islet deterioration. This study focused on 1 toxin-induced and 2 autoimmune rodent models of T1D: BioBreeding Diabetes Resistant rats, nonobese diabetic mice, and Dark Agouti rats treated with streptozotocin. Immunochemistry was used to evaluate the insulin levels in the **β**-cells, cell composition, and insulitis. T1D caused complete or significant loss of **β**-cells in all animal models, while increasing numbers of **α**-cells. Lymphocytic infiltration was noted in and around islets early in the progression of autoimmune diabetes. The loss of lymphocytic infiltration coincided with the absence of **β**-cells. In all models, the remaining **α**- and **δ**-cells regrouped by relocating to the islet center. The resulting islets were smaller in size and irregularly shaped. Insulin injections subsequent to induction of toxin-induced diabetes significantly preserved **β**-cells and islet morphology. Diabetes in animal models is anatomically heterogeneous and involves important changes in numbers and location of the remaining **α**- and **δ**-cells. Comparisons with human pancreatic sections from healthy and diabetic donors showed similar morphological changes to the diabetic BBDR rat model.

## 1. Introduction

Rodent models of diabetes are frequently used in basic science and in industrial environments, such as the pharmaceutical industry. Animal models of diabetes have been used for the past 150 years and were instrumental in the discovery of insulin [1]. In humans with type 1 diabetes (T1D), it is estimated that 70% of the pancreatic *β*-cell mass has been destroyed by the time clinical signs of the disease are present [2]. Without safe methods of sampling or visualizing the human endocrine pancreas, animals are essential models of the disease. Rodent models have enabled the discovery of key scientific findings, but frequently these findings do not translate to the clinical setting [2]. 

The common rodent models of T1D include the BioBreeding Diabetes-Resistant (BBDR) rat, the nonobese diabetic (NOD) mice, and the streptozotocin-induced diabetic rodents. Rodents studied for T1D can be broadly classified as having either spontaneous or inducible forms of the disease. In spontaneous diabetes, such as the NOD mouse, the genetic background results in a defined prevalence of the disease [2]. In contrast, with inducible diabetes, the disease is precipitated by exposure to defined antigens or reagents [2]. While the endpoint of hyperglycemia is the same, the path to eventual diabetes is quite different in each case.

The NOD mouse is an inbred strain that spontaneously develops autoimmune diabetes similar in pattern and genetic susceptibility loci to human T1D [1, 3]. Although it has been around for over 30 years, it still remains a pillar of diabetes research [4] with over 8000 publications utilizing the model. Interestingly, NOD mice housed in pathogen-free facilities exhibit an increased incidence of diabetes relative to those housed in conventional facilities [3]. 

The original BioBreeding rat colony was established in Canada in the 1970s. Fifty percent of the rats from the original line developed diabetes spontaneously. Since human clinical data suggested that diabetes was associated with environmental factors such as viral exposures [5–7], a virally inducible diabetic rat was a goal of selective breeding, resulting in the BBDR rat [8, 9]. The BBDR rat has many features that resemble human diabetes including development of hyperglycemia in a predictable manner, progression of insulitis, and susceptibility to other autoimmune diseases [9].

Streptozotocin (STZ) is a nitrosourea analogue widely used to induce experimental diabetes in rodents, because it is thought to have little extrapancreatic toxicity. Streptozotocin is a donor of nitric oxide, which is known to be destructive to pancreatic islet cells (reviewed in [10]). In addition, STZ produces superoxide anions due to its action on the mitochondria [10]. The drug causes structural changes in the pancreatic *β*-cells, including significant degranulation within 48 hours after administration [11]. Although it is assumed to have few nondiabetic side effects, it has been shown to alter the metabolism of other drugs [11]. 

While it is known that these animal models develop diabetes and respond to treatment differently [12], there have been few studies that compared the 3 animal models with regards to changes in the pancreatic islets with the progression of the disease and how they compare to human T1D. The focus of this study was to compare islet morphology and lymphocyte infiltration between three commonly utilized rodent models of T1D. Two models are classified as autoimmune models (BBDR rat and NOD mouse), and one was toxin induced (streptozotocin-injected rat). Both the BBDR and the streptozotocin-treated rats are inducible, meaning that the researcher can induce diabetes in some animals and leave others as the matched controls. In contrast, the NOD mouse develops diabetes spontaneously.

## 2. Materials and Methods

### 2.1. Diabetes Induction and Monitoring 

#### 2.1.1. Induced Autoimmune Diabetes: BioBreeding Diabetes Resistant (BBDR) Rat

Thirty-two male BBDR rats (Biomedical Research Models Inc., MA) of age 23–25 days were used. The rats were divided into 2 groups: 10 nondiabetic controls (C) and 22 diabetics (D). Induction of diabetes was performed as described earlier [13]. Briefly, the diabetic group rats were injected with the anti-RT6 monoclonal antibody DS4.23 hybridoma supernatant (2 mL/day for 5 days/week; kindly provided by Dr. Dale L. Greiner, University of Massachusetts Medical Center). Rats were also injected with a nonspecific immune system activator polyinosinic-polycytidylic acid (Poly I : C, Sigma, St. Louis, MO; 5 *μ*g/g body mass, 3 days/week). Poly I : C activates toll-like receptors on islets, leading to activation of a proinflammatory pathway that initiates the innate immune response. Control animals were injected with vehicle. After confirmation of increased plasma glucose levels (≥200 mg/dL) for 3 consecutive days, the rats were considered diabetic. Since diabetic BBDR rats cannot survive without insulin, an osmotic pump filled with insulin was implanted subcutaneously on the back of each rat with the flow rate of insulin delivery at 0.25 *μ*L/hr, predetermined by the manufacturer (ALZET, Cupertino, CA). The pumps lasted 4 weeks; therefore, for the remaining time of the study, rats were injected with insulin manually. Blood glucose levels (nonfasting) were measured every two days using a digital glucose meter AccuCheck Active (Roche Diagnostics, Indianapolis, IN). Animals were sacrificed after 8 weeks of diabetes to ensure that maximal changes in islets were achieved. 

#### 2.1.2. Spontaneously Developed Autoimmune Diabetes: Nonobese Diabetic (NOD) Mouse

Twelve 8-week-old female NOD/ShiLtJ mice were purchased from Jackson Laboratories (Bar Harbor, ME) and housed individually in barrier cages. Blood glucose levels were measured periodically using a OneTouch Ultra 2 glucometer (LifeScan Inc., Milpitas, CA) with animals being fasted for 2 h before each measurement. Mice were considered diabetic after two consecutive readings >250 mg/dL. Body weight was recorded weekly. Control mice were those who did not spontaneously develop diabetes. Mice were sacrificed at 23 weeks of age.

#### 2.1.3. Rat Model of Chemically Induced Diabetes: Streptozotocin (STZ) Induced Diabetes

Nine male Dark Agouti (DA) rats (10 weeks old) were randomly assigned to 3 groups: nondiabetic control (C), STZ-treated diabetic normoglycemic (DN), and STZ-treated diabetic hyperglycemic (DH). DA rats were chosen because they have no genetic inbreeding that makes them susceptible to diabetes; yet, they are an inbred strain. Diabetes was induced in the former 2 groups by single intraperitoneal STZ injection (65 mg/kg of body weight). Blood glucose levels were measured daily after STZ injections. When blood glucose levels reached >11.11 mM/L, rats were considered diabetic. Six days after STZ injections, insulin pellets (2 units/day) were inserted subcutaneously into the DN rats in order to maintain normal blood glucose levels. The duration of diabetes was 8 weeks at the time of sacrifice. Blood glucose levels (nonfasting) were monitored in all rat groups for the entire duration of diabetes using a digital glucose meter AccuCheck Active. 

### 2.2. Human Pancreas Source

Human pancreas samples from two male donors were purchased from Axon Cells (Keswick, VA). Tissue donors had the following description. Subject 1 served as a control with no history of diabetes, high blood pressure, or other chronic conditions. He was 37 years old with a BMI of 28.8. Subject 2 had been diagnosed with T1D for 26 years and was 40 years old, with a BMI of 23.5. Subject 2 had been treated with NovoLog x4/day and Lantus every 8 hrs at the time of death. Tissue arrived fixed in 10% normal buffered formalin and was embedded in paraffin after arrival. Tissue sectioning and staining were completed as described later.

### 2.3. Tissue Harvesting and Preparation

BBDR and DA rats were euthanized with an overdose of sodium pentobarbital. NOD mice were euthanized using cervical dislocation. Pancreata were removed and fixed in 4% paraformaldehyde in phosphate buffered saline (PBS), pH 7.2, for three days at +4°C. Tissue was embedded in paraffin using an automated vacuum tissue processor Leica ASP300S (Leica Microsystems Inc., Bannockburn, IL) and stored at +4°C. Tissue sections of 8 *μ*m thickness were cut using a microtome RM2255 (Leica Microsystems Inc.) and mounted directly on Superfrost/Plus microscope slides (Fisher, Pittsburgh, PA, no. 12-550-12). After cutting, slides were dried at +40°C overnight in an oven and stored at +4°C until processing.

Paraffin embedded sections were deparaffinized/rehydrated in xylene followed by ethanol and PBS serial rehydration. Antigen retrieval was completed in a steamer using 0.01 M citrate buffer, pH 6.2, with 0.002 M EDTA, for 30 min. After cooling for 20 min, slides were washed in PBS 2 times and permeabilized in 1% Triton X-100 in PBS for 30 min. Slides were rinsed again in PBS. After washing, sections were encircled with a PAP pen. Sections were incubated in 10% normal donkey serum (NDS), 1% bovine serum albumin (BSA), and 0.03% Triton X-100, all diluted in PBS, for 30 min to block nonspecific binding sites and rinsed in PBS. Blocked sections were used for immunofluorescence (IF) and immunohistochemistry (IHC) staining.

### 2.4. Immunofluorescence (IF)

Blocked sections were incubated with the primary antibody mix at +4°C, overnight, in a wet chamber. Sections were rinsed in PBS 3 times and incubated for 2 hr at room temperature in a mix of fluorophore conjugated secondary antibodies in a dark wet chamber. The following solution was used to dilute primary and secondary antibodies: 1% NDS, 1% BSA, and 0.03% Triton X-100. After incubation with secondary antibodies, slides were washed in PBS 3 times and mounted with antifading agent Gel/Mount (Biomeda, Foster City, CA). In some cases, DAPI (4′6-diamindino-2-phenylindole; 0.5 *μ*g/mL; Molecular Probes, Eugene, OR, no. D1306) staining was performed for 5 min at room temperature following the first wash after secondary antibody exposure.

The following primary antibodies were used to stain the pancreas: anti-insulin (1 : 200, Abcam, Cambridge, MA, no. ab7842) or anti-insulin (1 : 100,Santa Cruz Biotechnology, Inc., Santa Cruz, CA, no. sc-9168), antiglucagon (1 : 300, Abcam, no. ab10988), antisomatostatin (1 : 300, Abcam, no. ab53165), and anti-Ki67 Proliferation Marker (1 : 200, Abcam, no. ab16667). Appropriate secondary antibodies were used that were conjugated with DyLight 488 (1 : 400, Jackson ImmunoResearch Laboratories Inc., West Grove, PA, no. 706-485-148), Alexa 555 (1 : 400, Molecular Probes, Eugene, OR, no. A31570), or Alexa 647 (1 : 400, Molecular Probes, no. A31573).

Images were captured on a Nikon C1Si or C1 Plus confocal microscopes (Nikon Instruments Inc., Melville, NY). IF images were analyzed using Nikon software EZ-C1 3.90 Free viewer. The cellular composition of islets was measured by counting the individual types of cells (*β*-cells labeled with anti-insulin, *α*-cells with antiglucagon, and *δ*-cells with antisomatostatin) in each islet and dividing the number of each cell type by the total number of all labeled cells per islet.

### 2.5. Immunohistochemistry (IHC)

Anti-insulin (1 : 100, Santa Cruz Biotechnology, Inc., Santa Cruz, CA, no. sc-9168) or anti-glucagon (1 : 200, Santa Cruz Biotechnology, no. sc-13091) primary antibodies were used. Staining was developed using Histostain-*Plus* Broad Spectrum (AEC) Kit (Invitrogen, Frederick, MD, no. 859943). The IHC procedure was conducted according to manufacturer instructions. Slides were counterstained with hematoxylin to identify cell nuclei.

After staining, slides were rinsed in deionized water and placed on coverslips in Clear Mount mounting medium (Electron Microscopy Sciences, Hatfield, PA, no. 17985-12). The specificity of immunoreactivity was confirmed by omitting the primary antibody from some sections. The staining was observed using a light microscope Nikon Eclipse 80i (Nikon Instruments Inc., Melville, NY). Images were analyzed using Ps Adobe Photoshop CZ4 extended software. The relative insulin content was measured based on the intensity of staining of pancreatic sections with anti-insulin. The average pixel value of staining per cell or per islet was determined. Background staining was subtracted from each value. Cellular hypertrophy was defined as an increase in cell surface area that is greater than 25% above the mean surface area in cells from controls.

Insulitis was determined by the presence of lymphocyte infiltration, which was defined as highly concentrated monocytic nuclei around islets. Infiltration was scored using images of hematoxylin staining combined with IHC with either insulin or glucagon antibody labeling. Islets were scored using the following criteria: peri-insulitis when infiltration had begun with peripherally observed immune cells; intrainsular insulitis when immune cells had clearly infiltrated the islet; the islet destruction stage was determined when the islet area was completely infiltrated by immune cells. Infiltration was calculated as the percentage of the islet area comprised of infiltrating cells.

### 2.6. Statistics

For all experiments with more than 2 groups, one-way ANOVA on ranks (Kruskal-Wallis) followed by Dunn's pairwise comparisons was used. A *t*-test was used to compare total insulin content as determined by insulin immunoreactivity. For the other immunostaining experiments, nested ANOVA was used. All figures include means ± SEs. *P* value, defined as <0.05, was considered statistically significant. 

## 3. Results 

### 3.1. BioBreeding Diabetes Resistant (BBDR) Rat

**Figure 1:**
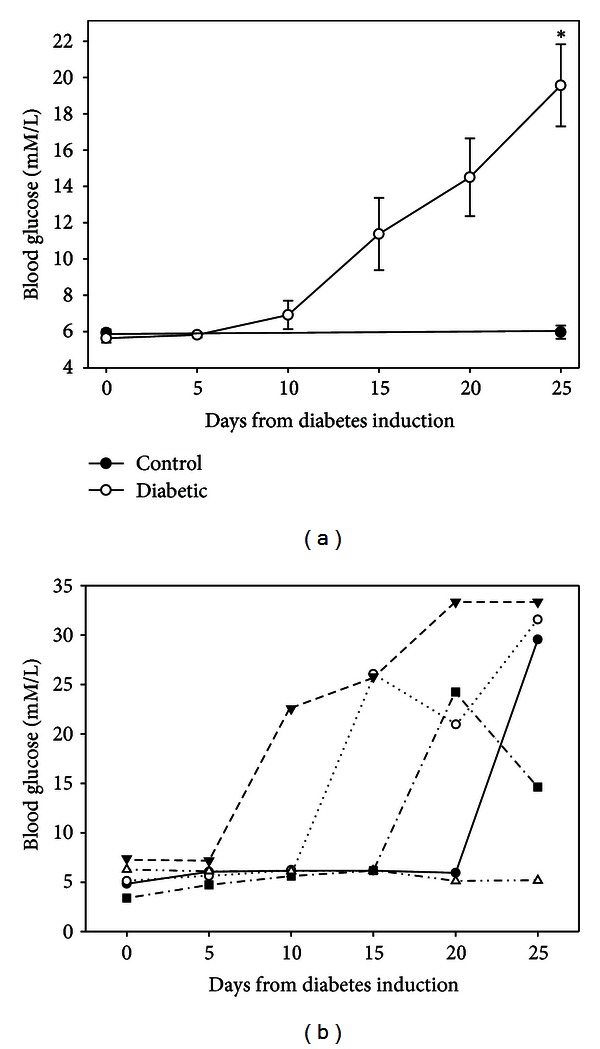
Blood glucose levels of BBDR rats. (a) Following induction of diabetes, average blood glucose levels gradually increased over the next 25 days but remained constant in controls (* = *P* < 0.001). (b) In analyzing the progression of diabetes in the individual animals, the onset of hyperglycemia varied by 15 days. Each line follows the blood glucose level for an individual rat chosen to demonstrate the variation in diabetes induction.

BBDR rats have an inducible form of autoimmune diabetes that must be initiated when the animals are 21–28 days old. Because of their young age, they continue to grow during the progression of diabetes, as shown in Table 1. Both the control and diabetic animals gained over 200% of their starting body weight and there was no statistical difference between groups. Blood glucose levels rose steadily over the 3 weeks following induction of diabetes (Figure 1(a)). Yet at the individual animal level, there was great variation in the onset of diabetes (defined as blood glucose >11.1 mM/L for 2 consecutive days).  Figure 1(b) provides examples of some of the variations in the onset of hyperglycemia. 

**Figure 2:**
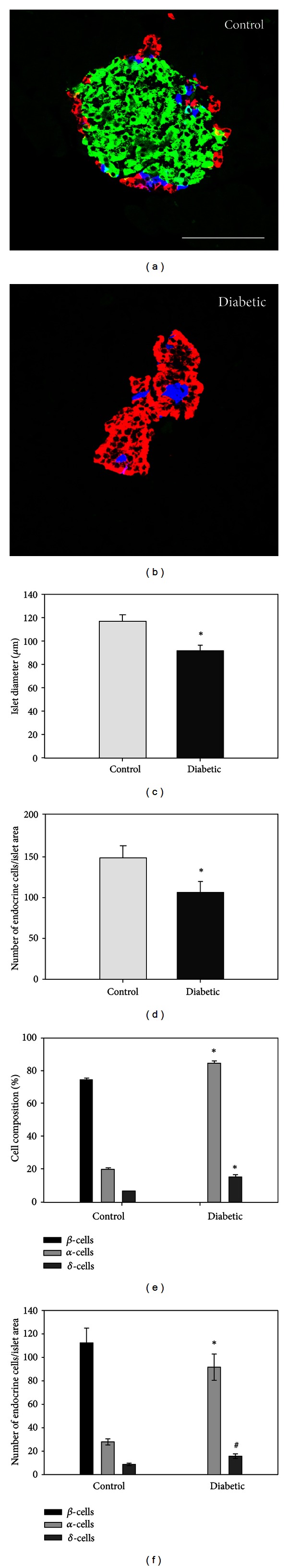
Islet size and composition in BBDR rats. (a) Immunofluorescence staining of an islet from a control animal with a *β*-cell core (anti-insulin; green staining), surrounded by *α*-cells (antiglucagon; red), and *δ*-cells (antisomatostatin; blue). Scale bar = 100 *μ*m. (b) Same staining protocol used on an islet from a diabetic animal (scale the same). (c) Islet diameter decreased in animals with diabetes (* = *P* < 0.001). (d) This decrease was not due to cell atrophy but to a loss in the number of cells (* = *P* < 0.005). (e) The cell composition, as measured by the percentage of endocrine cells, was dramatically different for the 2 groups. Islets of control animals were predominantly *β*-cells, while diabetic animals' islets were predominantly *α*-cells (*P* < 0.001). (f) The change in cell composition was due to a decrease in the actual number of *β*-cells and a simultaneous increase in the number of *α*-cells and *δ*-cells (* = *P* < 0.001; ^#^ = *P* < 0.005). *N* = 62 islets from 3 control animals and 79 islets from 3 diabetic rats for all graphs.

Islets were immune-stained for 3 major pancreatic hormones: insulin, glucagon, and somatostatin. They displayed an oval shape in control rats and irregularly shaped islets in the diabetic animals (Figures 2(a) and 2(b)). The average islet diameter from diabetic animals was 22% less than control (Figure 2(c)). To rule out the possibility that the loss in islet size was due to the atrophy of individual cells, cell numbers per cross-sectional area were counted. There were fewer total endocrine cells (*α*-, *β*-, and *δ*-cells) in the islets from diabetic animals (Figure 2(d)).

In addition to changes in islet size and cell number, the cellular composition was altered by diabetes. In control BBDR rats, *β*-cells were plentiful and found in the core of the islet, with *α*- and *δ*-cells on the mantle (Figure 2(a)). In contrast, in 79 islets analyzed from diabetic animals, no *β*-cells were identified (Figure 2(b)). Of interest, the remaining endocrine cells were predominantly *α*-cells (Figure 2(e)). Since the total islet cell number decreased with diabetes, it was unclear if the number of *α*-cells remained constant or increased as *β*-cells were destroyed due to diabetes. Thus, the number of individual *α*- and *δ*-cells were counted in sections from the same 79 islets. Islets from diabetic animals had 3.3-fold more *α*-cells and 1.8-fold more *δ*-cells compared to controls per islet cross-section (Figure 2(f)). The total increase of combined number of *α*- and *δ*-cells in islets was 2.9-fold from diabetic animals compared to controls. There were no detectible dividing cells in the diabetic islets when stained with the proliferation marker, Ki67 (results not shown).

Observation of diabetic islets revealed that the *δ*-cells were organized in small clusters surrounded by *α*-cells. On average, each cluster contained 9 *δ*-cells and was surrounded by at least two layers of *α*-cells. The ratio between *δ*- and *α*-cells in the islet was 9 to 51. Large islets from diabetic animals showed several *δ*-cell clusters (Figure 2(b)), while small islets sometimes consisted of one cluster only.

**Figure 3:**
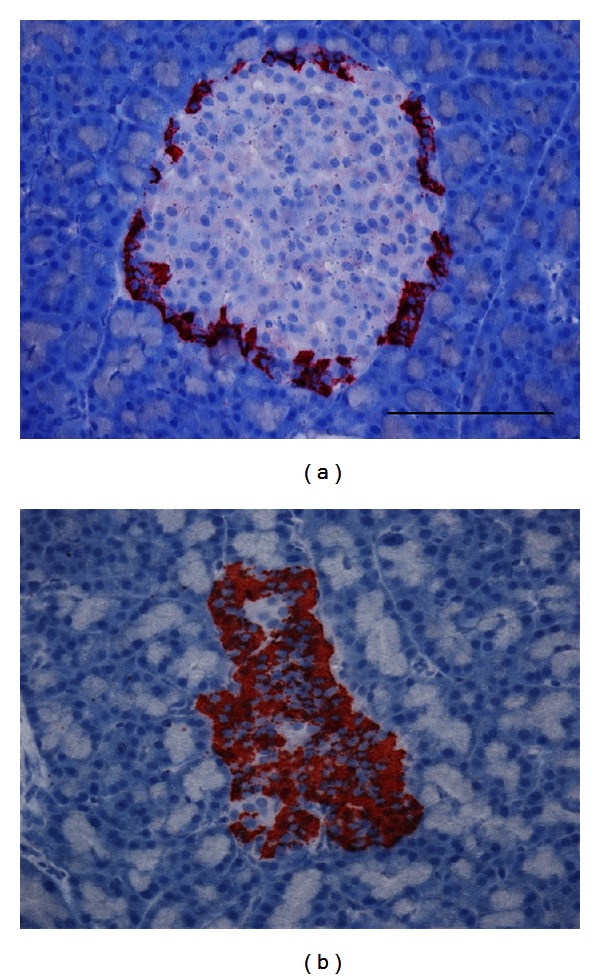
Lymphocyte infiltration in BBDR rat pancreas. Staining of *α*-cells with anti-glucagon antibodies (red), counter-stained with hematoxylin, showed no immune cell infiltration in the control (a) or diabetic (b) rats. Scale bar = 50 *μ*m.

Since no *β*-cells were detected in islets from diabetic animals by IF staining, glucagon antibody was used to label *α*-cells to identify islets in diabetic animals. Both IHC and IF illustrated a rim of *α*-cells in the islets from nondiabetic controls. In combination with hematoxylin staining, calculations of insulitis in the rats were conducted (Figure 3). Surprisingly, no insulitis was detected in any of the diabetic animals (Figure 3(b)) or in controls (Figure 3(a)). Our inability to detect insulitis coincided with the observation that no *β*-cells remained in the islets at the time of sacrifice.

### 3.2. Nonobese Diabetic (NOD) Mouse

**Figure 4:**
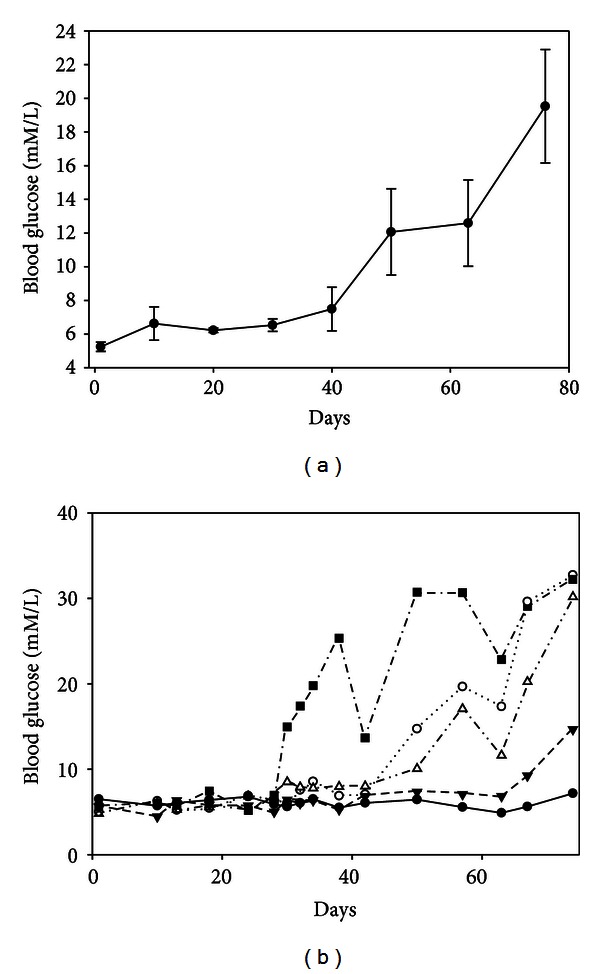
Blood glucose of NOD mice. (a) Average blood glucose levels gradually increased over an 80-day window in NOD mice. (b) In analyzing the progression of diabetes in the individual animals, there was wide variation in the onset of hyperglycemia, including some animals that did not develop hyperglycemia. Each line follows the blood glucose level for an individual mouse chosen to demonstrate the variation in diabetes induction.

Female NOD mice were used as a model of spontaneously developed autoimmune diabetes. There was no difference in the starting body weight of animals that were diabetic and those that failed to spontaneously develop the disease (Table 1), nor was there any difference in their ending weight or % change. Blood glucose levels were monitored from birth until week 23 when animals were sacrificed. Average blood glucose levels rose steadily over the course of the study (Figure 4(a)). However, there was wide variation in the onset of diabetes, and some of the animals failed to become diabetic (Figure 4(b)), which is typical of the NOD model. 

**Figure 5:**
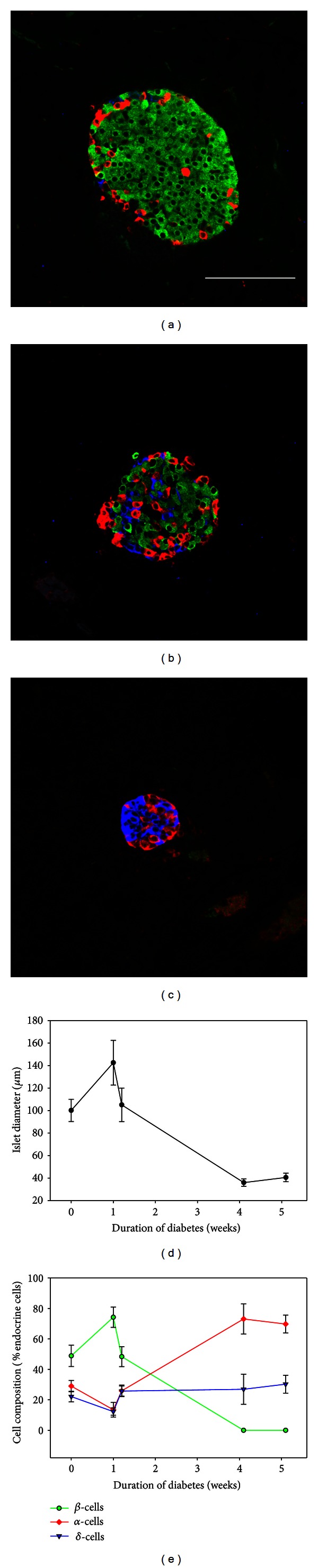
Islet size and cell composition in NOD mice. (a) Islets from NOD mouse that did not develop hyperglycemia were immunostained for *β*-cells (green), *α*-cells (red), and *δ*-cells (blue). Scale bar—100 *μ*m for all images. (b) Islet from an NOD mouse diabetic for 1 week. (c) Islet from an NOD mouse diabetic for 3 weeks. (d) Because the onset of hyperglycemia was extremely variable in the NOD mice, islet diameter was plotted according to the duration of diabetes. Islet diameter decreased with increasing duration of diabetes. (e) Cell composition was also plotted according to the duration of hyperglycemia. With increasing duration of diabetes, *β*-cells were lost and *α*-cell numbers increased. *N* for both graphs = 11.803 cells from 116 islets from 6 mice.

Figure 5 illustrates the differences in islet size between NOD mice that failed to develop diabetes (Figure 5(a)) and those that had been diabetic for 1 (Figure 5(b)) or 3 weeks (Figure 5(c)). While the average islet diameter was 100 *μ*m, there was great variation among animals corresponding to the duration of diabetes. Thus, the average islet diameter was plotted according to the duration of diabetes (Figure 5(d)). As the duration of the disease progressed, the islet size decreased. 

Triple IF staining determined that islets composed of the 3 types of cells (*β*-, *α*-, and *δ*-cells) were found in mice that did not develop diabetes (Figure 5(a)) or had a duration of diabetes of less than 10 days. At an early stage of diabetes (1 week), the normal glucagon-somatostatin rim was absent, and *α*- and *δ*-cells were found at the center of the islet (Figure 5(b)). As the disease progressed, the *β*-cells numbers decreased. The majority of islets remaining after 4 weeks of diabetes were small (approximately 50 *μ*m in diameter) (Figure 5(d)) and consisted of predominantly *α*-cells (Figure 5(e)). Similar to the BRDD rats, when large complex endocrine structures were identified, they were comprised of multiple *δ*-cell clusters surrounded by *α*-cells. In animals with a duration of diabetes of more than 4 weeks, insulin producing *β*-cells were completely absent (Figures 5(c) and 5(e)). 

**Figure 6:**
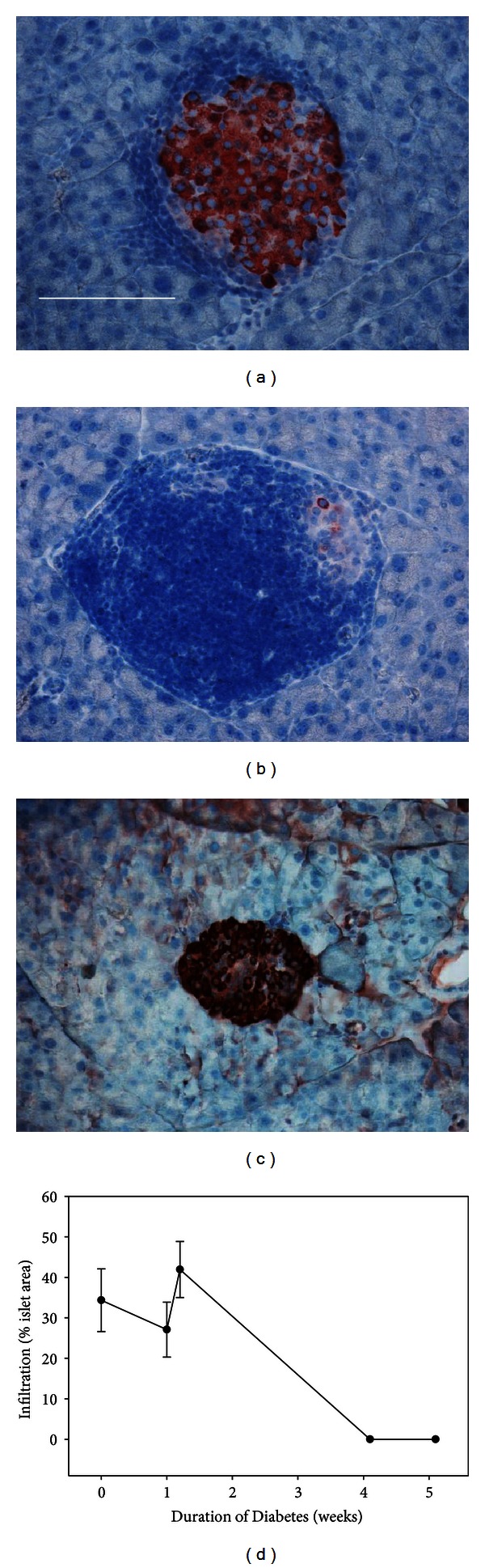
Lymphocyte infiltration in NOD mice. Staining of *β*-cells with anti-insulin antibodies, counter-stained with hematoxylin, revealed different levels of immune cell infiltration in mice that failed to develop diabetes, ranging from little infiltration (a) to complete penetration (b). The infiltrates are identified by a high density of blue nuclei surrounding the *β*-cells (red). Scale bar = 50 *μ*m for all images. (c) There was no infiltration noted at the late stages of diabetes. These sections had to be stained with antiglucagon rather than anti-insulin, because there were no insulin-positive cells at later time points. (d) A plot of the amount of infiltration per duration of diabetes summarizes the findings (*N* = 65 islets from 6 mice).

The intensity of the insulin immunolabeling (Figure 6(a), red), an indication the insulin content/*β*-cell [14, 15], was not different during the early stages of diabetes (less than 10 days duration). Average insulin intensity staining was as follows: controls 126 ± 10, 7 days diabetes 141 ± 4, and 9 days 150 ± 11. After 2 weeks of diabetes, there were no insulin-positive cells remaining to measure. 

Hematoxylin staining showed changes in the amount of insulitis with the duration of diabetes. Mice that did not develop diabetes had large variations in the amount of lymphocytic infiltration in individual islets (Figures 6(a) and 6(b)). In mice that developed diabetes, as the duration of diabetes increased, the amount of infiltration decreased to 0.  Figure 6(c) illustrates the lack of infiltration in a glucagon-labeled islet. Glucagon was used because there were no insulin-positive cells remaining in the islet at that time point. Those islets that had no remaining *β*-cells also had no signs of active lymphocyte infiltration (Figure 6(d)). 

### 3.3. STZ Induced Diabetes

Male DA rats, used as a model of toxin-induced T1D, were randomized into 3 groups: 2 STZ injection group and 1 control group. Blood glucose levels exceeded 11.11 mM/L after injection in all STZ-injected rats and continued to rise. After developing overt diabetes, half of the diabetic animals were implanted with insulin pellets to maintain their glucose levels in normal ranges (diabetic normoglycemic; DN group). The other group of STZ-treated rats was left hyperglycemic (diabetic hyperglycemic; DH group). 

**Figure 7:**
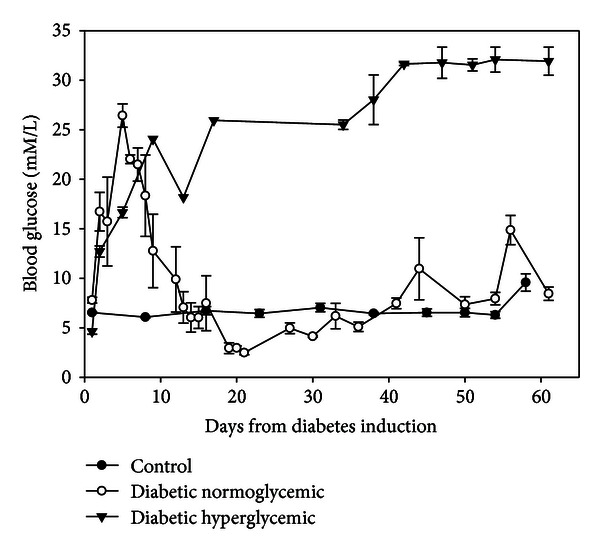
Blood glucose levels of STZ-treated DA rats. Diabetes was induced in DA rats by a STZ injection while 3 control animals were injected with vehicle. Blood glucose levels increased rapidly in the STZ-treated rats, both diabetic normoglycemic (DN) and diabetic hyperglycemic (DH). In the insulin treated rats (DN group), the blood glucose returned to normal values by day 12 (diamond symbol) following the insulin pellet implantation. Blood glucose levels of control animals were constant at approximately 110 mg/dL.

Rats in the untreated diabetic (hyperglycemic) group gained less weight when compared to control and diabetic normoglycemic groups (Table 1). As demonstrated in Figure 7, rats in the DH group had persistent hyperglycemia and rats in the DN group that were treated with insulin had blood glucose levels near control values. DH rats had increased levels of ketones in the blood when compared to the DN and control rats (3.3 ± 1.1, 0.4 ± 0.0, and 0.4 ± 0.1 mM/L, resp.). No ketones were present in the urine of the control rats, with categories of trace or small amounts in the DN rat group and trace to moderate levels in the DH group. 

**Figure 8:**
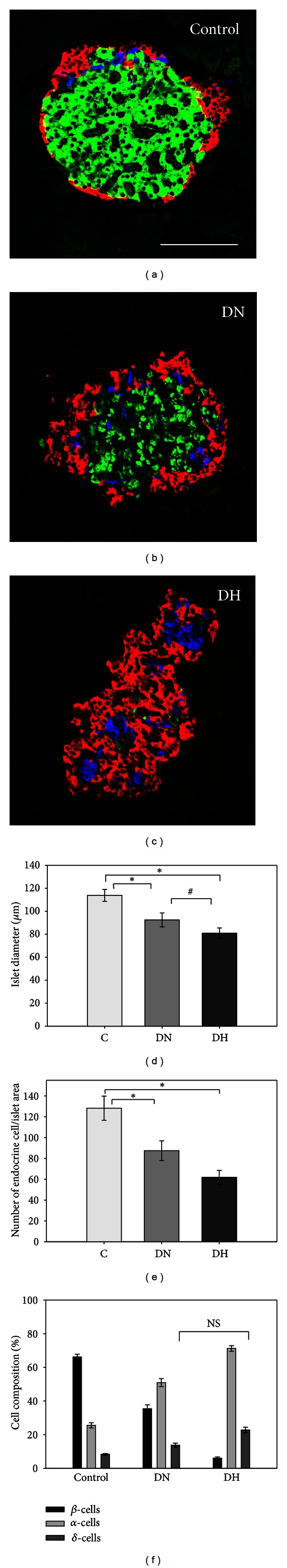
Islet size and cell composition in STZ-treated rats. (a) An islet from nondiabetic DA rat was large with a normal cellular morphology. Scale bar = 100 *μ*m for all images. (b) A representative islet from a normoglycemic STZ-treated rat was smaller, but still maintained the *β*-cell core (green) with the *α*- (red) and *δ*-cells (blue) rim. (c) Typical islet from hyperglycemic rat was smaller than the other 2 groups with few *β*-cells. (d) Islet diameter was plotted, and each group was more statistically different than the others. (e) The number of cells per islet area was less in the 2 STZ-treated groups (DN and DH) compared to controls. (f) Cell composition was plotted showing a loss of *β*-cells with hyperglycemia. Cell composition from each of the 3 groups was different for each cell type except for the *δ*-cells between the DN and DH groups (NS = not significant). *N* of islets = 96 from 3 controls, 73 from 3 DN rats, and 74 in the DH group (3 rats).

Eight weeks after the STZ injection, islet size and structure were investigated based on antibody labeling. Islet size significantly decreased in both STZ-treated animal groups, DN and DH (Figures 8(a)–8(c)). Islets from rats in the DN group had a 19% smaller diameter compared to controls, and the islet diameter for the DH group was 30% less than controls (Figure 8(d)). In addition, the number of individual endocrine cells per islet decreased in the 2 diabetic groups compared to controls (Figure 8(e)).

Islets from the control (Figure 8(a)) and DN (Figure 8(b)) groups displayed the typical islet architecture with an *α*- and *δ*-cell mantle, covering a *β*-cell core. A further loss of *β*-cells was noted in the DH group with irregular shaped islets without an *α*- and *δ*-cell rim (Figure 8(c)). Hyperglycemic and normoglycemic diabetic animals had fewer *β*-cells than control rats, but unlike diabetic NOD mice and the BBDR rats, they did not lose all of their *β*-cells. Importantly, insulin treatment attenuated the *β*-cell loss in the DN group when compared to those animals with extremely high blood glucose levels, the DH group (Figure 8(f)). Among the 3 rat groups, the composition of cells was statistically different for each cell type, with the exception of *δ*-cells between the 2 STZ-treated groups (Figure 8(f)).

**Figure 9:**
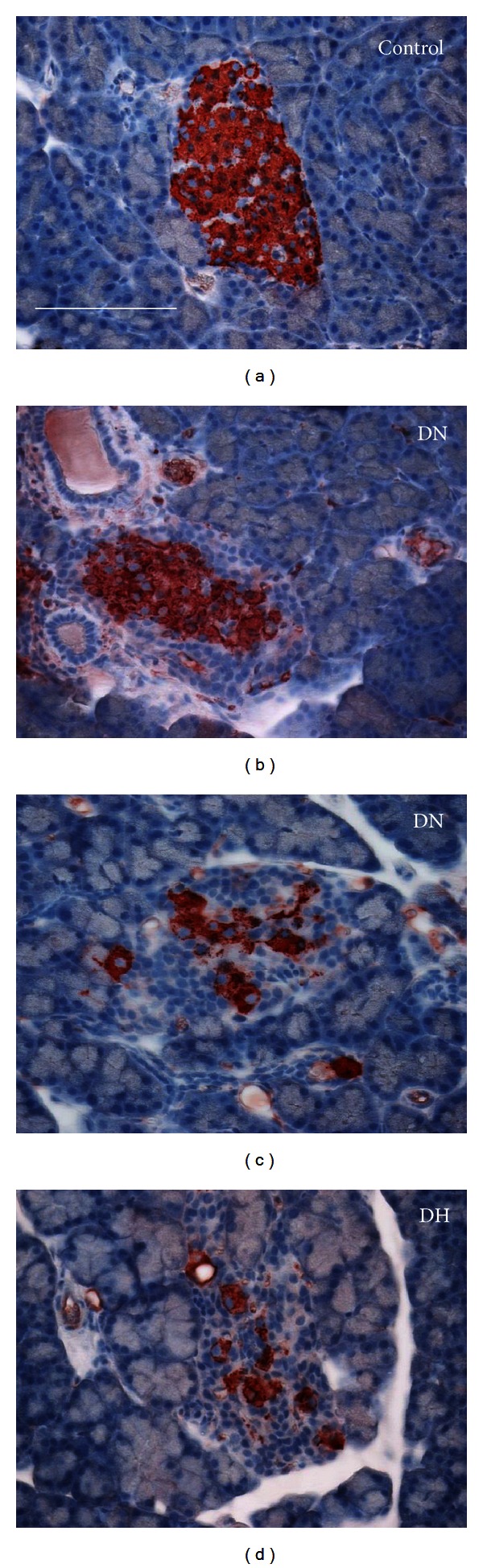
Lymphocyte infiltration in STZ-treated rats. DA rat pancreas was labeled with insulin antibody and with hematoxylin to reveal nuclear staining. (a) Insulin positive islets showed even staining in control rats. Scale bar = 100 *μ*m for all images. (b) Diabetic normoglycemic (DN) rats showed weak insulin staining in the islet core with atrophied *β*-cells. (c) There were also islets with scattered hypertrophied *β*-cells in the DN group. (d) Diabetic hyperglycemic rats showed few hypertrophied *β*-cells.

When stained for insulin, control rat pancreata revealed intense staining in the islets (Figure 9(a)). Diabetic normoglycemic (DN) rats had 3 different subtypes of islets. First, islets with weak insulin staining in the core, representing atrophied *β*-cells comprised 15% of all islets (Figure 9(b)). The pixel intensity representing insulin content was statistically lower in these *β*-cells than in islets from control animals (*P* < 0.05; 150 ± 1) pixel values for controls (*N* = 76 islets) and 120 ± 2 for atrophied cells from diabetics (*N* = 43 islets). Second, islets with scattered hypertrophied beta cells comprised 5% of all islets (Figure 9(c)). These islets had a greater insulin immunostaining intensity of 165 ± 2 compared to controls (*N* = 43, *P* < 0.05). The majority of islets (80%) from the DN group had a combination of both atrophied and hypertrophied *β*-cells (not shown). Insulin-positive cells in the diabetic hyperglycemic group were predominantly hypertrophied *β*-cells (Figure 9(d)) with an insulin intensity of 164 ± 1 (*N* = 99 islets). 

### 3.4. Human Pancreatic Islet Investigations

**Figure 10:**
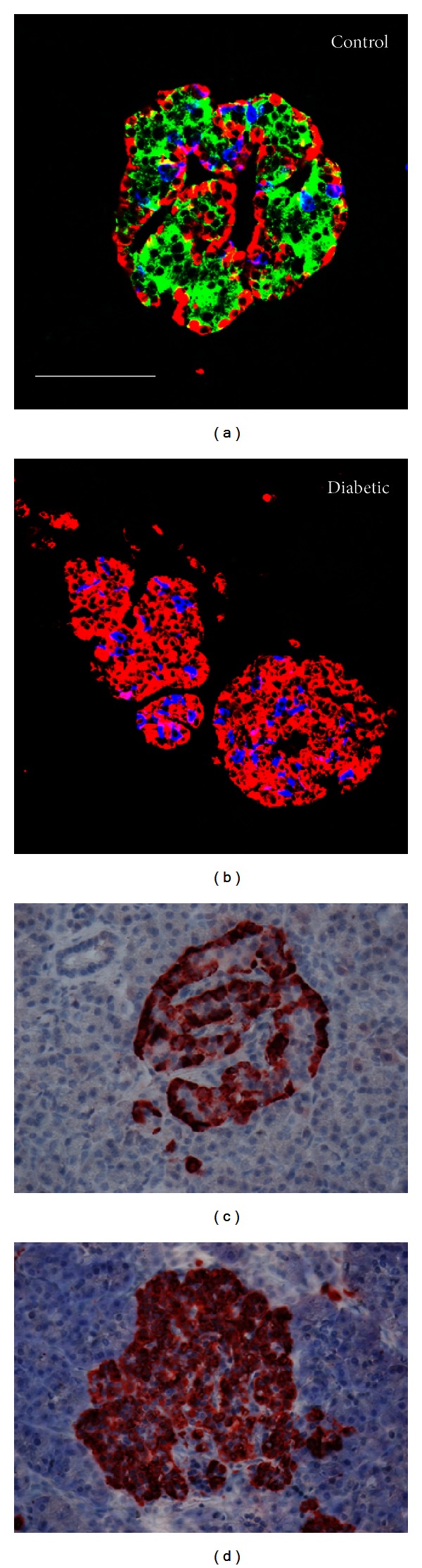
Human islet morphology and infiltration. (a) An islet from nondiabetic human donor was large with a normal cellular morphology. Scale bar = 100 *μ*m for both images. (b) An example of an islet from a donor with controlled diabetes showed a lack of *β*-cells. (c) Glucagon staining illustrates the lack of infiltration in the healthy donor. (d) The donor with long-standing diabetes also had no signs of infiltration.

Figure 10(a) shows a typical human islet from a nondiabetic donor. Unlike the rodent models, the *α*- and *δ*-cells are scattered throughout the islet, while still maintaining somewhat of a rim appearance of *α*-cells. A majority of the islets from the diabetic donor were composed of *α*- and *δ*-cells (Figure 10(b)). In fact, only 1 of 25 islets analyzed had remaining *β*-cells. The diabetic pancreatic islets varied in size and shape, ranging from 20 cells to large oblong islets up to 400 *μ*m in diameter. Of interest, single *β*-cells were detected in the pancreas of the diabetic patient, but not the control. They were hypertrophied and often sickle shaped (not shown). Similar to the rodent models, no signs of infiltration were noted in any of the 40 islets examined from the healthy control (Figure 10(c)) or the diabetic donor (Figure 10(d)) as judged by glucagon IHC in combination with hematoxylin labeling and confirmed by insulin immune-staining (not shown).

## 4. Discussion

Rodent animal models are widely used to study diabetes both to understand the pathogenesis and to investigate potential therapeutic treatments. In the NOD mouse, over 200 therapies have been shown to prevent or reverse diabetes [16]; yet, none of these have translated to therapies that prevent or cure T1D in humans [2, 17]. That may be due to differences in the autoimmunity associated with humans and rodents, but it may also be due to the differences in islet morphology and function when comparing rodent and human islets [18, 19]. An understanding of these differences is essential to target meaningful therapies that will translate into the clinic.

The progression of diabetes, as measured by blood glucose levels, is correlated with islet health. Our study compares changes in the islet cell structure and composition among three different animal models of T1D. While previous studies have focused on the invading cells that surround the islets and the corresponding islets changes early in the onset of the disease [20, 21], the current work focuses on the long-term outcome on the islets for each diabetic rodent model. Analysis of pancreatic islets in autoimmune animal models (BBDR rat and NOD mouse) showed complete loss of insulin producing *β*-cells. In all three examined models, including the STZ-injected rat, the loss of *β*-cells caused an expanded population of *α*- and *δ*-cells, which were organized with clusters of *δ*-cell cores surrounded by *α*-cells. When compared to a human with a long history of treated T1D, the complete *β*-cell loss was again apparent, with an invasion, and likely expansion, of *α*-cells. This increase in density and number of *α*- and *δ*-cells in islets of humans with diabetes and in STZ-treated rats was identified 35 years ago by Unger [22]. The new findings provided in this study illustrate that only the rodent islets, and not human, were reorganized in consistent patterns of *δ*-cells surrounded by *α*-cells, and even though it was clear that the *α*-cell numbers had increased with disease progression, no proliferating cells were positively stained with the Ki67 proliferation marker. We hypothesize that *β*-cell loss, *α*-cell expansion, and infiltration all occur within the same period, and when the *β*-cells are finally depleted, not only does the infiltration subside, but the *α*-cell proliferation halts as well. 

Most interesting was the sparing of *β*-cell loss in the STZ-treated rats. Even with a long duration of uncontrolled diabetes, the hyperglycemic rats still had clearly identifiable *β*-cells within the islets. However, it is important to note that *β*-cell destruction in response to STZ is dose dependent. Thus, at higher doses all *β*-cell would likely have been eliminated. In addition, alloxan is an appropriate substitute for STZ that likely would have provided the same dose-dependent results. 

The survival of *β*-cell in our STZ-treated rats helps to explain how the STZ rats, while extremely hyperglycemic, were able to survive without insulin treatment for weeks. When animals were treated with insulin to maintain normal blood glucose levels, significantly more *β*-cells were spared. While *α*- and *δ*-cell numbers did increase with both diabetes groups, the greatest change was in the hyperglycemic STZ-treated animals. By analogy, serum from some humans with T1D has c-peptide reactivity, suggesting the presence of a small number of *β*-cells [23]. 

When studying the autoimmunity of T1D, the BBDR rats and the NOD mice are the preferred models. The first histomorphological change detectable in the BB rat pancreas is the infiltration of immune cells into the islets [9]. It has been suggested in both of these models (BBDR and NOD) that *β*-cell apoptosis begins when the animals are still neonates [24]. Our findings of infiltration early in the progression of diabetes in NOD mice are supported by previous reports. At around 3 weeks of age, lymphocytic infiltration has been measured [25], with full-blown infiltration at 4-5 weeks of age [4]. Our work shows that infiltration is already occurring before hyperglycemia is measured, in support of previous studies [2, 26]. Unique to this study was the finding that when the *β*-cells were destroyed in the 2 autoimmune models, no infiltrating lymphocytes remained. This was the case in both the BBDR rats and the NOD mice and was consistent with the human samples. 

## 5. Conclusions

Three rodent models of T1D were studied after lengthy bouts of diabetes and were compared to a pancreatic sample from a human with long-standing T1D. While each model has advantages and disadvantages, including the investigator's ability to control the onset of diabetes with inducible models, and autoimmunity that is reminiscent of the human form, each also has limitations that must be considered when designing experiments. For interventions aimed at sparing *β*-cells, the moderate-dose STZ model used here may be superior, because there are a few *β*-cells that still remain and appear to function after a long duration of diabetes. This study illustrates great heterogeneity between islets in the same animal model, and thus conclusions must be based on analysis of a large number of islets. Perhaps most important, lymphocytic infiltration was only noted early in the duration of diabetes coinciding with the existence of *β*-cells. Together, these findings stress the importance of choosing the appropriate animal model for the hypothesis to be tested.

## Figures and Tables

**Table 1:** 

Body weight (gm)	Starting weight controls	% Change	Starting weight diabetic	% Change	Diabetic normoglycemic	% Change
BBDR rats	112 ± 5	203 ± 47	128 ± 3	211 ± 10	—	—
NOD mice	25 ± 1	0	24 ± 1	−2 ± 0	—	—
STZ-treated rats	217 ± 6	26 ± 4	216 ± 3	7 ± 1*	241 ± 7	26 ± 2

*P  < 0.05.
